# A New Approach Integrating Brood-Associated Semiochemicals with Additional Feeding for Honey Bee (*Apis mellifera*) Colony Development

**DOI:** 10.3390/insects17030294

**Published:** 2026-03-07

**Authors:** Irina Ciotlaus, Ana Balea, Diana Klara Gaia, Maria Pojar-Fenesan

**Affiliations:** 1 Raluca Ripan Institute for Research in Chemistry, Babeș-Bolyai University, 30 Fântânele Street, 400294 Cluj-Napoca, Romania; irina.ciotlaus@ubbcluj.ro (I.C.); diana.molnar@ubbcluj.ro (D.K.G.); maria.fenesan@ubbcluj.ro (M.P.-F.); 2 Faculty of Environmental Sciences and Engineering, Babeș-Bolyai University, 30 Fântânele Street, 400294 Cluj-Napoca, Romania

**Keywords:** fatty acids, behavioral role, nutrition, *Apis mellifera*, queen oviposition stimulation

## Abstract

The aim of this study was to identify chemical formulations that stimulate *Apis mellifera* colony development by enhancing queen egg-laying under protein-supplemented conditions. Feeding trials were conducted in early spring, when natural food sources are scarce. The experiment was conducted in two Romanian apiaries and included four treatment groups. Three formulations included protein-enriched bee food: two standard variants and one supplemented with essential fatty acids. All were administered alongside behavioral stimulants (T1–T3). A fourth treatment served as a control, containing only protein-based food without brood pheromones or additional stimulants (T4). Pheromone blends were formulated based on brood-emitted volatiles identified by solid-phase microextraction-gas chromatography coupled with mass spectrometry (SPME–GC–MS). The effects of the treatments were evaluated by measuring queen egg-laying and brood area development. Results showed that treatments based on brood ester pheromones (BEP)–T1 and a fatty acid blend (FAB)–T3 significantly stimulated queen egg-laying and brood production, with comparable performance and a slight advantage for T3. In contrast, combining BEP with a fatty acid-supplemented protein diet (T2) produced a moderate effect, consistent with regulated lipid intake in honey bee colonies. These findings indicate that brood-associated semiochemicals, combined with protein supplementation, can effectively stimulate colony growth.

## Simple Summary

Reductions in floral resources caused by climate change and agricultural intensification increasingly challenge honey bee colony development. Under these conditions, additional feeding and methods that stimulate colony activity are often needed to support colony growth. In this study, we evaluated the effects of brood-associated semiochemicals combined with additional feeding on the development of *Apis mellifera* colonies. Colonies receiving combined stimulation showed increased queen egg-laying, which led to improved brood production and overall colony development compared to untreated control colonies. This research contributes to a better understanding of the physiological and behavioral mechanisms that help maintain strong and resilient honey bee colonies.

## 1. Introduction

The global decline in bee populations has been partly attributed to reduced queen egg-laying performance, leading to diminish colony growth and disruptions in pollination-dependent ecosystems. The queen’s egg-laying rate is a critical determinant of colony development as increased reproductive activity directly supports colony expansion [1].

Nutrition plays an essential role in colony performance influencing queen fecundity and brood dynamics [2,3]. In periods of limited floral resources and unfavorable climatic conditions, beekeepers commonly apply supplemental feeding strategies to stimulate early spring development. Various nutritional interventions have been proposed to enhance colony vitality and brood production [4,5,6]. However, nutritional stimulation alone may produce variable results, as metabolic regulation and colony physiology are influenced by complex interaction between diet, worker activity, and brood presence [7,8].

Protein-based diets formulated with various pollen sources improve bee survival and brood rearing compared to non-pollen controls. Moreover, protein nutrition has been shown to interact with pheromonal signaling pathways, suggesting a link between dietary status and responses to chemical stimuli [9,10].

Chemical communication is fundamental to social insect organization, being involved in reproduction, nutrition, and defense against pathogens [11,12,13]. In the *Apis mellifera*, brood-derived semiochemicacals regulate worker behavior, physiology, and foraging dynamics [14]. As shown by Akongte et al., exogenous pheromone applications can rapidly modulate colony responses [15].

The SPME–GC–MS technique is commonly applied for profiling volatile organic compounds (VOCs) in the hive environment, including emissions from larvae, pupae, brood, adult bees [16,17,18], and apicultural products [19,20].

The increase in honeybee colony performance at the beginning of the beekeeping season is closely linked to stimulating the queen’s egg-laying and maintaining active nurse bees. During the nursing phase, the hypopharyngeal (HPGs) and mandibular glands (MGs) reach maximum development and secrete royal jelly (RJ), a process stimulated by brood presence and influenced by nutritional intake [21,22,23].

Brood ester pheromones (BEPs) consist of a blend of 10 methyl and ethyl fatty acid esters [24]. Acting as primer and releaser signals, BEPs modulate nurse bee physiology and behavior, stimulating royal jelly production, foraging activity, and queen egg-laying [25,26,27,28]. Field studies report enhanced colony vigor, pollination efficiency, and crop yields following BEP application [29,30]. Synthetic BEP formulations have also been associated with improved queen acceptance and reproductive performance, although their effectiveness depends on the availability of natural food resources [31,32,33].

While BEPs have been extensively studied for their regulatory role in colony behavior, increasing evidence suggests that free fatty acids may also contribute to chemical signaling within the hive. Certain fatty acids, such as oleic acid, are known to elicit specific behavioral responses, including hygienic activity [34]. Cuticular profiles, comprising hydrocarbons, fatty acids, their esters, and other waxy lipids, contribute to colony-specific odor and nestmate recognition [35,36,37,38,39]. In addition, fatty acids have been detected in brood-related matrices and are considered biologically active components involved in communication processes [40,41]. These observations support the hypothesis that free fatty acids may act as complementary or alternative semiochemicals influencing colony physiology.

BEPs are biosynthesized from specific fatty acid precursors through enzymatic esterification processes [24]. Considering that oleic, linoleic, and linolenic acids represent natural precursors of BEP components, it is reasonable to hypothesize that these free fatty acids may exert complementary or alternative stimulatory effects at the colony level. Moreover, previous studies have shown that the biological efficacy of brood pheromones depends on colony context and resource availability, highlighting a potential synergistic interaction between semiochemical stimulation and protein supplementation [33].

Therefore, the present study aimed to investigate whether free fatty acids, applied as exogenous stimuli under protein-supplemented conditions, can stimulate queen egg-laying and brood development in early spring, in comparison with a synthetic BEP formulation. The four experimental variants include different types of stimulation while maintaining the same protein base intake in order to avoid nutritional restriction and to enable a specific assessment of the effects of the semiochemical compounds.

The novelty of this study lies in investigating free fatty acids, not merely as nutritional components, but as potential semiochemical mediators capable of modulating colony reproductive dynamics.

## 2. Materials and Methods

### 2.1. Sample Collection

The experimental tests were conducted using a local strain of honey bee, predominantly based on the Romanian subspecies *Apis mellifera carpatica*. Honey bee brood samples were collected from the experimental apiary located in Suatu, Cluj County, Romania in early spring (March 2023). Brood combs containing larvae aged 7–9 days (L5 stage) were collected from the hives and stored at −20 °C until extraction. The experiments were conducted in two apiaries located in Cluj County, Romania: Apiary 1, situated in Suatu (SU) and Apiary 2, situated in Caianu (CA).

### 2.2. Chemicals

The following analytical-grade reagents (purity > 95%) were used: chloroform, methanol, ethanol, and petroleum ether (Sigma-Aldrich, St. Louis, MO, USA); KOH, NaCl, H_2_SO_4_, and anhydrous MgSO_4_ (Alfa Aesar, Thermo Fisher Scientific, Karlsruhe, Germany); linoleic and oleic acids (Merck, Darmstadt, Germany); linolenic acid (TCI, Tokyo Chemical Industry Co., Tokyo, Japan); stearic and palmitic acids (Sigma-Aldrich, USA). Artificial feed (solid protein food) was purchased from commercial suppliers and included sucrose, fructose, glucose, water, defatted soy flour, inactive brewer’s yeast, and pollen.

### 2.3. Volatile Compounds from Brood

#### 2.3.1. SPME Extraction

Volatile compounds were extracted using a 50/30 μm DVB/CAR/PDMS SPME fiber (Supelco, Bellefonte, PA, USA). Prior to use, the fiber was conditioned at 220 °C for 1 h in the injector port of an Agilent 7890 gas chromatograph (Agilent Technologies, Santa Clara, CA, USA). Each 20 mL headspace vial was filled with approximately 20 frozen L5 larvae (2.5 g), sealed with a PTFE septum, and equilibrated for 15 min at room temperature. The fiber was then exposed to the headspace for 30 min, followed by thermal desorption at 260 °C for 5 min in splitless mode. Each sample was extracted in triplicate.

#### 2.3.2. Solvent Extraction

Approximately 2.5 g of frozen L5 larvae were homogenized in a biphasic solvent system comprising 4 mL chloroform, 2 mL methanol, and 3 mL of 10% (*w*/*v*) aqueous NaCl. The mixture was stirred for 20 h, then centrifuged at 3000 rpm for 10 min. The organic phase was separated, dried over anhydrous MgSO_4_, filtered, and concentrated under a nitrogen stream. Extracts were stored at −20 °C until GC-MS analysis.

#### 2.3.3. GC-MS Analysis

Analyses were performed using a GC-MS system (Agilent 7890 GC with 5975 Series MSD, Agilent Technologies, Palo Alto, CA, USA) equipped with a DB-FFAP nitroterephthalic acid-modified polyethylene glycol column (30 m × 0.25 mm × 0.25 μm). For SPME samples (Section 2.3.1), the fiber was inserted directly into the injector (splitless mode). For solvent-extracted samples (Section 2.3.2), 1 μL of extract was injected. The injector was set at 250 °C. The oven temperature program was as follows: initial hold at 50 °C (1 min), ramp to 200 °C at 10 °C/min, then to 240 °C at 5 °C/min, held at 240 °C for 10 min. Helium served as the carrier gas at 1 mL/min flow rate. Mass spectrometry was performed in scan mode (*m*/*z* 50–500 a.m.u.) with 70 eV ionization energy. Data were processed using MSD ChemStation software version 2.0. The identification of volatile compounds was supported by the NIST library and further confirmed using linear retention indices (LRIs) calculated with a C8–C20 and C21–C40 alkane standards mixture (Sigma-Aldrich). Compound identification was based on the relative peak area percentages.

### 2.4. Synthesis of Methyl and Ethyl Esters of Fatty Acids

To synthesize the methyl and ethyl esters, 1 mol of a saturated or unsaturated fatty acid was reacted with 5 mol methanol or ethanol in the presence of 0.2 mol concentrated H_2_SO_4_ under magnetic stirring. The mixture was refluxed for 5 h. Reaction progress was monitored by thin-layer chromatography (TLC) on silica gel G (benzene: ether, 2:1), visualized using H_2_SO_4_ (d = 1.25). After cooling, the reaction mixture was poured over crushed ice and extracted with diethyl ether. The organic phase was washed successively with brine and aqueous NaHCO_3_ until reaching neutral pH, dried over anhydrous MgSO_4_, and concentrated. Yields of 70–75% were obtained, with GC purity ranging between 98 and 99%. The esters were analyzed using the Agilent 7890 GC and 5975 Series MSD, equipped with a HP-5MS (5%-phenyl)-methyl polysiloxane fused silica capillary column (30 m × 0.25 mm × 0.25 μm). The injector was set at 250 °C. The oven temperature program was as follows: initial hold at 50 °C (1 min), ramp to 210 °C at 2 °C/min (10 min), then to 300 °C at 5 °C/min, held at 300 °C for 5 min. Helium served as the carrier gas at 1 mL/min flow rate. Mass spectrometry was performed in scan mode (*m*/*z* 50–500 a.m.u.) with 70 eV ionization energy. Data were processed using MSD ChemStation software.

### 2.5. Preparation of Stimulatory Compounds

#### 2.5.1. Fatty Acid-Based Stimulant (FAB)

The fatty acid-based stimulant was formulated with the following mass composition: palmitic acid (7%), stearic acid (3%), oleic acid (18%), and linoleic acid (72%). To stabilize the mixture, 1% α-tocopherol was added. One milligram of the active mixture was dissolved in 0.05 mL dichloromethane and embedded into a textile matrix (4 × 7 cm). The impregnated fabric was sealed in a polyethylene sachet for storage until use.

#### 2.5.2. Synthetic Brood Ester Pheromone (BEP) Blend

The synthetic BEP blend was prepared with the following mass percentages: methyl palmitate (10%), ethyl palmitate (11%), methyl linoleate (5%), methyl linolenate (14%), methyl oleate (16%), ethyl oleate (3%), methyl stearate (15%), ethyl linoleate (4%), ethyl linolenate (17%), and ethyl stearate (5%). Dichloromethane was used as a solvent, and 1% α-tocopherol was added for stability. A 0.5 mg aliquot of the mixture (in 0.05 mL dichloromethane) was applied to rubber stoppers used for hive conditioning.

### 2.6. Controlled Release Studies

To ensure biologically relevant concentrations of the active stimulatory compounds throughout the experimental period, controlled-release strategies were implemented. Treated materials (e.g., fabric pads) were periodically replaced within the hive to maintain consistent volatile emission levels. The release profiles were monitored to confirm effective compound diffusion into the hive environment.

### 2.7. Experimental Setup and Organization

The experiments were conducted in two apiaries located in Cluj County, Romania: Apiary 1, situated in Suatu (46°50′ N, 23°58′ E), and Apiary 2, situated in Caianu (46°47′ N, 23°58′ E). The study period extended from March to April 2023, which corresponds to the early spring phase of increasing queen egg-laying activity, typically peaking in June. Optimal climatic conditions were targeted for the experiments: daily temperatures exceeding 15 °C, nighttime temperatures of at least 5–7 °C, and the absence of wind and precipitation.

This study analyzes hives with comparable configurations, including similar colony strength and an equivalent number of frames. The colonies were located in commercial apiaries managed by local beekeepers under standard beekeeping practices.

Each hive selected for the experiment contained comparable amounts of stored honey from the previous season, ensuring consistent baseline nutritional conditions.

The experiment involved four treatment groups, each consisting of three colonies per treatment, resulting in a total of 12 colonies per apiary. The limited number of treatment replicates (n = 3) resulted from logistical constraints associated with field-based experiments conducted in commercial beekeeping environments. In these settings, maintaining colony uniformity and adhering to standardized management practices restrict the practical scale of experimentation. Treatments were randomly distributed across the apiary field to avoid spatial bias.

The four experimental treatments are summarized in Table 1.

Egg-laying surface and brood area were quantified at each inspection using a frame count method based on a grid system, as described by Shaher et al. (2018) [42]. The occupied area on both sides of each frame was measured by counting square units and ex-pressed as surface area (dm^2^).

All treatments, including T4, received identical protein supplementation to prevent nutritional bias and to guarantee that any observed differences were due to the semiochemical compounds being tested.

### 2.8. Statistical Analysis

Statistical analysis and data representations were carried out using ORIGIN 2025 software (OriginLab, Northampton, MA, USA). Statistical analyses were performed separately for the two apiaries. Differences among treatments (T1–T4) for egg-laying and brood area were evaluated using one-way ANOVA, followed by Tukey’s Honestly Significant difference test (Tukey’s HSD test) when the overall model was significant (α = 0.05). Data are reported as mean ± SE, and treatments sharing the same letter were not significantly different. Pearson correlation coefficients were calculated to assess the relationship between egg-laying and brood area within each apiary.

## 3. Results

This study aimed to evaluate different spring feeding strategies to enhance honey bee (*Apis mellifera*) brood development, focusing on the bioactivity of synthetic semiochemical mixtures based on fatty acid-derived brood pheromones.

### 3.1. Volatile Organic Compounds Identified in Brood Samples

A series of volatile organic compounds (VOCs) were identified in brood samples using both SPME and solvent extraction followed by GC-MS analysis (Table 2).

Table 2 presents the volatile compounds extracted from 7–9-day-old larvae using SPME. A total of 15 compounds were identified, with linoleic acid (57.88%) and oleic acid (18.42%) being the most abundant components. Other major constituents included palmitic acid (5.62%) and stearic acid (3.82%), all of which are biologically relevant in the synthesis of brood pheromones. In the brood extract (Table 2), obtained using a methanol–chloroform extraction system, 37 compounds were identified, including a wide range of saturated hydrocarbons, alkenes, fatty acids, and their methyl esters, such as palmitoleic, stearic, and linoleic acids, all present in minor but relevant amounts.

Both extraction methods revealed a consistent profile dominated by C16–C18 fatty acids and their esterified derivatives, compounds previously reported as brood-associated semiochemicals [24,41]. In our study, based on their abundance and documented biological relevance in brood signaling pathways [41,43], we selected a subset of saturated and unsaturated C16–C18 fatty acids for the feeding trials, aiming to evaluate their stimulatory effect on egg-laying and brood development.

The chemical signaling of the brood, via the continuous release of VOCs, is important to regulating colony physiology; therefore, pheromone stimulation treatments must be evaluated with regard to the natural signals emitted by the brood [44].

### 3.2. GC-MS Analysis of Synthetic Stimulatory Blends

To confirm the chemical integrity and composition of the synthetic stimulatory formulations used in the experimental treatments, GC-MS analysis was performed for both:

- The Synthetic Brood Ester Pheromone Blend (BEP);
- The Fatty Acid Stimulation Component Blend (FAB).

**Chemical Composition of BEP**

**Figure 1:**
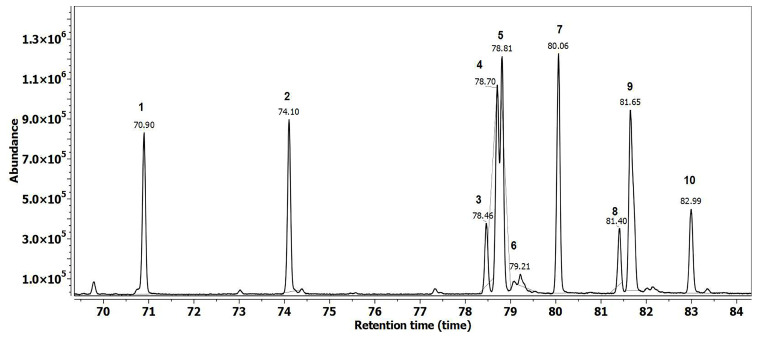
Gas chromatographic profile of the BEP synthetic pheromone formulation. Legend: 1. Methyl palmitate (70.90)—10%; 2. Ethyl palmitate (74.10)—11%; 3. Methyl linoleate (78.46)—5%; 4. Methyl linolenate (78.70)—14%; 5. Methyl oleate (78.81)—16%; 6. Ethyl oleate (79.21)—3%; 7. Methyl stearate (80.06)—15%; 8. Ethyl linoleate (81.40)—4%; 9. Ethyl linolenate (81.65)—17%; 10. Ethyl stearate (82.99)—5%.

Figure 1 displays the total ion chromatogram of the synthetic BEP blend used in experimental treatments. The blend was formulated based on naturally occurring brood pheromone components, with methyl and ethyl esters of C16–C18 fatty acids.

The composition of the synthetic BEP mixture obtained in this study was qualitatively comparable to that of the natural brood pheromone described by Le Conte et al. (1990) [24], consisting mainly of methyl and ethyl esters of palmitic, oleic, linoleic, linolenic, and stearic acids. The synthetic mixture formulated in the present study reproduced the same qualitative profile, with minor adjustments to the proportions to facilitate experimental formulation and application.

This BEP formulation is part of treatments T1 and T2 and was used to evaluate its biological effects on bee colonies in a comparative study with treatments T3 and T4.

**Chemical Composition of FAB**

**Figure 2:**
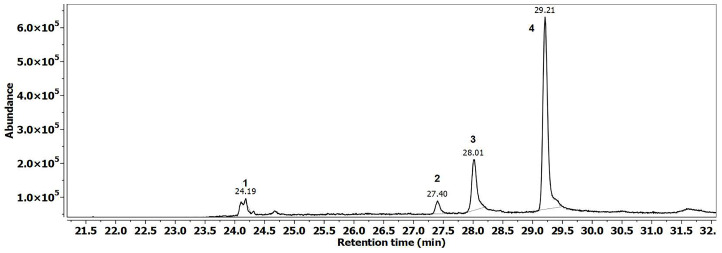
Gas—chromatographic profile of the FA stimulatory component. Legend: 1. Palmitic acid (24.19)—7%; 2. Stearic acid (27.40)—3%; 3. Oleic acid (28.01)—18%. 4. Linoleic acid (29.21)—72%.

Figure 2 shows the GC–MS chromatogram of the fatty acid blend (FAB), representing the stimulatory component used in Treatment 3.

The composition of mixture T3 (FAB) was established based on the volatile compounds identified in the brood extracts (Table 2), where by C16–C18 fatty acids were consistently present and have been indicated in the literature for their role in larval chemical communication. These acids are also natural precursors to the esters that make up the brood ester pheromone (BEP). We aimed to evaluate the potential stimulatory effect of free fatty acids on brood-associated behaviors by including them in a distinct formulation, compared to the specific esters in the BEP.

In treatment 3, linoleic acid comprised the majority of the mixture at 72%, followed by oleic acid at 18%, palmitic acid at 7%, and stearic acid at 3%. This formulated mixture was then impregnated into a textile dispenser, which was subsequently utilized in apiaries to assess its biological effects on bee colonies.

This formulation was tested in the field to determine whether direct supplementation with key fatty acids could elicit physiological or behavioral responses in nurse bees and promote increased egg-laying by the queen.

**Relevance of Chemical Profiles to Bioactivity**

Both synthetic blends reflected the chemical composition of natural brood extracts, matching the volatile fatty acids and brood pheromone components identified in Table 1. The presence of C16–C18 fatty acids, especially linoleic and oleic acids, confirms the chemical fidelity of the formulations and supports their biological relevance.

These GC–MS results validate that the synthetic stimuli are consistent with natural larval semiochemicals, forming the basis for the subsequent field tests presented below.

### 3.3. Controlled Release System for Fatty Acid Stimulant

The fatty acid blend (FAB) mixture used for colony stimulation was applied onto a textile matrix designed to act as a controlled-release dispenser. The formulation was stabilized by incorporating 1% α-tocopherol, which helps prevent oxidative degradation of the unsaturated fatty acids.

Each dispenser consisted of 1 mg of active substance impregnated into a 4 × 7 cm textile support and sealed in a polyethylene bag until deployment. Upon introduction into the hive, the dispenser was unsealed to allow volatilization of active components into the colony environment.

To ensure a consistent and biologically relevant concentration of the volatile fatty acids throughout the trial period, the dispensers were:

- Weighed before and after each 7-day interval to monitor compound release;
- And replaced weekly with fresh dispensers.

Considering the well-known low volatility of fatty acids, the system was designed as a qualitative controlled-release method, based on weekly replacement of dispensers with identical chemical load.

### 3.4. Biological Response of Colonies to Experimental Treatments

Following the application of the experimental stimulants, weekly assessments were conducted to monitor queen egg-laying activity and brood development. Two key biological indicators were recorded:

- The surface area of combs with freshly laid eggs (dm^2^);
- The total brood area (dm^2^) per colony at the end of the experimental period.

Data were collected from four treatment groups (T1–T4), each consisting of three colonies, over a period of 4 to 6 weeks.

At the onset of the experiment (22 March), colonies in both apiaries (Suatu—SU and Căianu—CA) exhibited differences in their biological development status. This variability reflects the fact that the study was conducted under real beekeeping conditions. Consequently, certain initial differences between treatments were observed in the egg-laying surface and brood area. These baseline variations should therefore be interpreted in the context of natural colony heterogeneity.

#### 3.4.1. Biological Response in Apiary 1 (SU)

Table 3 represents the mean values (±SD) for egg-laying and the brood area observed across the four treatments (T1, T2, T3, and control T4) from 22 March to 20 April 2023, in Apiary 1 (SU). These indicators assess the queen’s reproductive efficacy and the colony’s growth potential.

**Figure 3:**
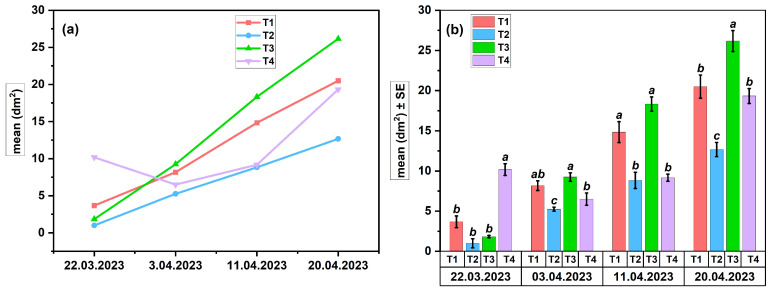
Egg-laying in colonies from Apiary 1 (SU): (**a**) temporal dynamics across treatments; (**b**) treatment effects (ANOVA, *p* < 0.05), letters indicating significant differences.

**Figure 4:**
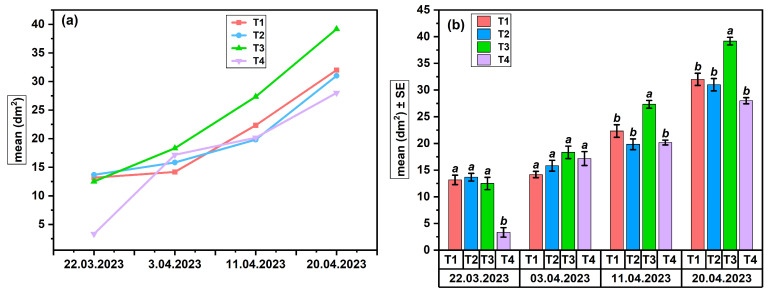
Brood area in colonies from Apiary 1 (SU): (**a**) temporal dynamics across treatments; (**b**) treatment effects (ANOVA, *p* < 0.05), letters indicating significant differences.

The results summarized in Table 3 are presented graphically in Figure 3 and Figure 4 to highlight the dynamics of egg-laying and brood development. The significance of the differences between treatments was statistically evaluated using ANOVA (*p* < 0.05).

**Egg-laying dynamics in Apiary 1**

The reproductive response of colonies in the Suatu apiary was evaluated by monitoring egg-laying dynamics, depending on the applied pheromone treatments and the associated protein nutritional diet.

The evolution of the average daily egg production (dm^2^) showed a progressive increase throughout the four-week experiment for all treatments (T1–T4), with an intensification of reproductive activity after the second week (Figure 3a). Treatments T1 and T2, which included brood ester pheromone (BEP) components, induced a steady but moderate increase compared to T3, which generated an increase in average values toward the end of the period. In contrast, witness (T4) showed an irregular variation, without a clear stimulation trend. The assessment of variance (ANOVA, *p* < 0.05, one-way, n = 3) indicated significant differences between treatments for all experimental data (F_(3,8)_ = 9.80–48.68, *p* < 0.01, R^2^ = 0.79–0.95) (Figure 3b). At the beginning of this study (22 March), the egg-laying surface (EL) was elevated at T4 (a), with no significant variations observed between the pheromone treatments (T1–T3, b) (F = 33.87, *p* < 0.001, R^2^ = 0.93). In the next stage (3 April), the values between treatments were comparable, with no significant differences (F = 2.89, *p* = 0.10, R^2^ = 0.52). Furthermore, T3 significantly surpassed T2 and T4, remaining at higher values until the end of the study. In the assessments on 11 April and 20 April, T3 showed the highest values (a), T1 had an intermediate response (ab, b), and T2 and T4 remained at lower levels (b, c). (F = 15.39–25.39, *p* < 0.01, R^2^ = 0.85–0.90). The detailed *p*-values corresponding to the data presented in Figure 3 are provided in Supplementary Table S1.

**Brood area development in Apiary 1**

In the Suatu apiary (Apiary 1), the average values (dm^2^) of the brood area showed a progressive increase over the 4 weeks of monitoring (Figure 4a,b).

The evolution of treatments (Figure 4a) indicates a constant increase in the brood surface area across all groups, with the most extensive growth observed in treatment T3, followed by T1 and T2, while T4 maintained lower values throughout the period.

Statistical ANOVA assessment (Figure 4b) revealed significant differences between treatments on 22 March (F(_3,8_) = 12.42, *p* < 0.01, R^2^ = 0.82), with T1–T3 having larger brood areas than T4. On 3 April, there were no differences between treatments (all: a; F(_3,8_) = 2.89, *p* = 0.102). On the contrary, on 11 April and 20 April, the T3 treatment significantly surpassed the other variants (*p* < 0.05; T3: a; T1, T2, T4: b), thereby confirming the sustained positive benefits of pheromone stimulation on brood development and the biological consistency of the colonies’ response over time. The pairwise comparisons underlying the differences presented in Figure 4 are detailed in Supplementary Table S2.

The results show high treatment efficacy of T3 in stimulating brood development compared to T1 and T2, which included BEP-type components and BEP associated with FA, respectively. The expansion of the brood area reflects more intense reproductive activity, associated with more abundant egg-laying and more dynamic development of the treated colonies. All treatments showed a progressive increase in brood surface area, with variations in amplitude between treatments and higher values compared to the control group.

#### 3.4.2. Biological Response in Apiary 2 (CA)

The application of the experimental protocol was extended to the Caianu apiary (Apiary 2), maintaining the same conditions and treatments used in Apiary 1 (Suatu), and the mean values (mean ± SD) for the egg-laying parameters and brood area are presented in Table 4.

**Egg-laying dynamics in Apiary 2**

**Figure 5:**
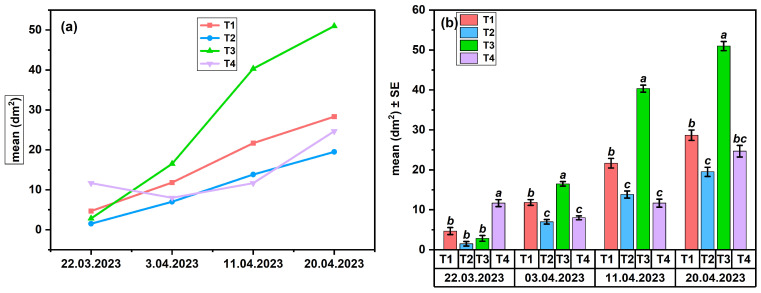
Egg-laying in colonies from Apiary 2 (CA): (**a**) temporal dynamics across treatments; (**b**) treatment effects (ANOVA, *p* < 0.05), letters indicating significant differences.

Figure 5a,b illustrate the temporal evolution of egg deposition.

At the beginning of the study (22 March), control group T4 recorded an average of 11.67 dm^2^, significantly higher than T1 (4.67 dm^2^), T2 (1.50 dm^2^), and T3 (2.83 dm^2^) (F = 33.87; *p* < 0.001; R^2^ = 0.93). In the following weeks, the values increased progressively in all treatments, but T3 recorded the highest values at the end (≈40.33 and 51.0 dm^2^), followed by T1 (≈28.67 dm^2^), while T4 had intermediate levels, above T2 (≈19.50 dm^2^). In Figure 5b, the letters indicate significant differences between the variants (T3 “a”, T1 “b”, T4 “bc”, T2 “c”). These data confirm the greater efficacy of the T3 pheromone treatment in stimulating egg-laying. The results of Tukey’s HSD pairwise comparisons associated with Figure 5 are reported in Supplementary Table S3.

**Brood area development in Apiary 2**

**Figure 6:**
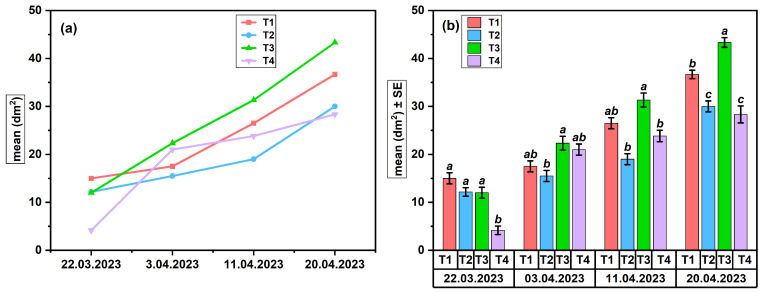
Brood colonies from Apiary 2 (CA): (**a**) temporal dynamics across treatments; (**b)** treatment effects (ANOVA, *p* < 0.05), letters indicating significant differences.

The temporal evolution of the brood area in the colonies at the Căianu apiary is illustrated in Figure 6a,b.

At the beginning of the period (22 March), the control group (T4) had significantly lower values in comparison to the pheromone treatments (F = 20.51, *p* < 0.001). In the following weeks, the brood area progressively expanded across all variants, with treatment T3 continuously showing the highest values, followed by T1, although T2 and T4 demonstrated lower levels. Significant differences across treatments occurred from 3 April (F = 6.45, *p* < 0.05) and subsequently intensified (F = 17.06–30.07, *p* < 0.001), with T3 correlating with group “a”, differentiated from the other variants (Figure 6b). The results demonstrate efficient pheromonal stimulation of brood development in T3, with a modest response in T1 and decreased responses in T2 and T4. Detailed *p*-values for Tukey’s HSD pairwise comparisons are provided in Supplementary Table S4.

#### 3.4.3. Comparative Analysis Between Apiary 1 and 2

**Figure 7:**
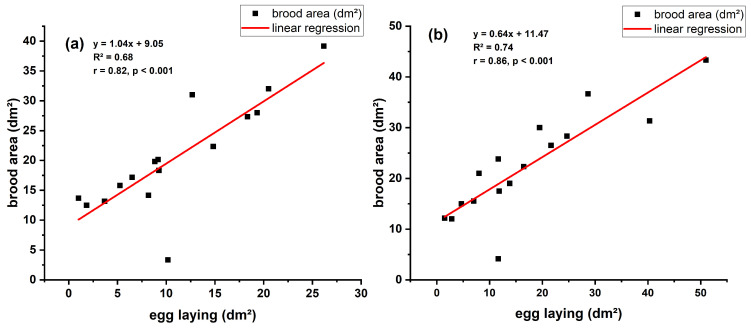
Relationship between egg-laying and brood area in colonies from two apiaries: (**a**) Apiary 1 (SU); (**b**) Apiary 2 (CA). Both sites showed a significant positive correlation (*p* < 0.001), with slightly stronger association in Apiary 2 (r = 0.86, R^2^ = 0.74) compared to Apiary 1 (r = 0.82, R^2^ = 0.68).

The Pearson correlation between egg-laying and brood area was positive and significant in both apiaries (Figure 7a,b). In Apiary 1 (SU), the correlation coefficient was r = 0.82 (*p* < 0.001), and in Apiary 2 (CA), r = 0.86 (*p* < 0.001), indicating a strong association between egg-laying intensity and brood area. The R^2^ values (0.68 and 0.74) suggest that 68–74% of the variation in brood area is explicable by the egg-laying rate, whereas the residual variability is attributed to biological differences between colonies and environmental conditions.

**Figure 8:**
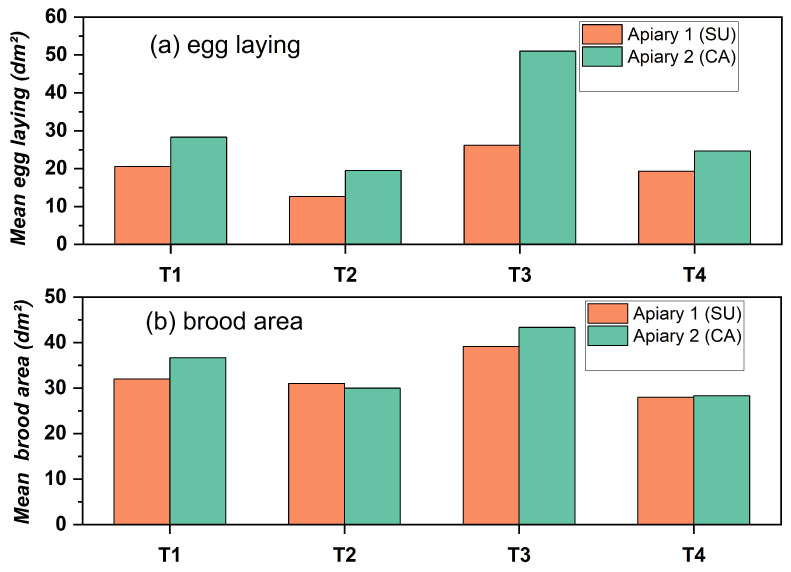
Egg-laying (**a**) and brood area (**b**) in colonies from Apiary 1 (SU) and Apiary 2 (CA) under treatments T1–T4 at the final sampling date (20 April 2023).

The mean results for egg deposition and brood area at the final assessment (20 April 2023) are illustrated in Figure 8a,b.

In both apiaries, treatment T3 exhibited the greatest values, followed by treatment T1, while T2 and T4 showed lower levels. Colonies in the CA apiary had higher values compared to those in the SU for both egg-laying and brood areas, signifying more vigorous development at this site. The prevailing pattern in the two apiaries substantiates the enhanced efficacy of the T3 pheromone treatment in promoting reproductive activity and brood development.

## 4. Discussion

This study compared the BEP formulation (Figure 1) with the FAB mixture derived from fatty acids identified in FAB larvae (Figure 2) to assess their efficacy of stimulating oviposition and early larval development under spring protein feeding conditions. Brood pheromones are extensively recognized as both a primer and releaser signal, influencing the physiology of nurse bees and the intensity of their feeding and grooming behaviors [25,41,43]. Prior research has demonstrated that the application of BEP can enhance egg-laying, accelerate colony growth, and improve harvesting efficiency [29,45].

In the Suatu apiary (Apiary 1), egg-laying (Figure 3a,b) exhibited the most rapid response to treatments, demonstrating greater sensitivity to pheromone stimulation than the brood area (Figure 4a,b), which includes both egg-laying and larval development success. Statistical data indicate that T3 (FAB) exhibited the strongest stimulatory effect, which was succeeded by T1 (BEP), but T2 and T4 showed significantly weaker responses. Despite an initially lower egg-laying intensity in T2, the brood area was comparable to that of the more active treatments, indicating a positive biological effect of protein feeding combined with FA. Bees exhibit increased sensitivity to the omega-6/omega-3 ratio and the overall concentration of fatty acids, with even a little imbalance in intake potentially leading to a temporary decline in colony performance [46,47]. Studies show that elevated concentrations of certain fatty acids in the diet influence reproduction and brood development [48]. A different hypothesis claims that dietary supplementation with fatty acids may influence metabolic homeostasis and the intestinal microbiome, as evidenced by studies on high-fat diets in bees [49]. The pheromone treatments (T1 and T3) significantly impacted the colonies, but the control group (T4) exhibited irregularities without a clear pattern of stimulation. A protein diet synergistically enhances pheromonal stimulation, promoting the growth of the hypopharyngeal glands for an optimal physiological response. The reduced effectiveness of the fatty acid diet (T2) compared to the dispenser treatment (T3) may stem from differences in how signals are perceived. Pheromonal cues, which are detected through olfactory pathways, can trigger quick physiological responses at the colony level. In contrast, dietary lipids influence the colony through slower processes related to digestion and metabolism [50].

The treatment dynamics at the Caianu apiary (Apiary 2) were predominantly similar to those recorded in Apiary 1. The egg-laying assessment (Figure 5a,b) indicates that T3 sustained the highest values from the first weeks. A particular aspect of Apiary 2 is that there were no major differences between treatments during the initial period (22 March–3 April), which shows a more homogeneous response from the colonies, possibly linked to their initial structure and favorable local conditions for early development. The distinction in the stimulating effects became visible at the final stage of the studies. The results regarding the brood surface area (Figure 6a,b) show that T3 is distinguished by the highest values, followed by T1 and T2, but T4 consistently maintained the lowest levels.

The results demonstrate that the tested pheromone variants had a stronger stimulatory effect on the queens’ reproductive response, evidenced by a quick increase in egg-laying activity. The brood area represents an integrated biological process that combines the effects of egg-laying and larval development success, which are influenced by factors such as hive temperature, protein dietary quality, and nurse bee activity. The positive outcomes observed in this study align with findings indicating that brood pheromones, BEPs, may accelerate colony growth, enhance brood area, and improve winter survival [30,42,51]. According to the comprehensive findings of Peso & Barron (2014) [33], the colonies’ reaction to BEP is contingent upon biological context, including colony status, resource availability, and seasonal factors, clarifying the differences noted among treatments and among apiaries.

Regression analysis (Figure 7) reveals a robust positive association in both apiaries, evidenced by high correlation coefficients (r = 0.82 and r = 0.86, *p* < 0.001), signifying that fluctuations in brood area may be predicted based on egg-laying intensity. The regression analysis indicates subtle variations among apiaries regarding the effectiveness of transforming egg-laying into actual brood, potentially reflecting microclimatic characteristics, familial composition, or colony management practices.

Approaching the final stage of the trial (Figure 8), the pheromone treatments produced similar outcomes in both apiaries, with T3 consistently exhibiting the highest rates of egg-laying and total brood area. T1 and T2 produced moderate reactions, whereas T4 exhibited low levels, consequently confirming the robustness of pheromone stimulation regardless of location. The stimulatory effects were more evident in Apiary 2, where the colonies exhibited a more uniform initial level of development, facilitating a better differentiation of treatments throughout the season.

The results obtained in the present study indicate that pheromonal stimulation, combined with a properly formulated protein diet, can generate a synergistic effect on queen activity and colony development. Carbohydrates primarily provide metabolic energy but do not supply the amino acids required for royal jelly synthesis and therefore are not the main limiting nutritional factor for colony reproduction. Protein nutrition is a key determinant of brood production and queen oviposition [4].

The present study has limitations due to the small number of colonies (n = 3), spatial variations between apiaries, and the short period of monitoring. Subsequent studies will cover a larger sample size, multi-seasonal monitoring, and an assessment of the processes via which pheromones affect colony development across varied environmental conditions. Another possible direction to explore is to determine the role of the lipidic component in the treatment, considering that certain fatty acids, such as oleic acid, are involved in necrophoric signaling and can stimulate hygienic behaviors in colonies [34].

This lipid formulation may have synergistically improved the efficacy of T3, offering a combined effect of the pheromone and hygiene stimulus. Nonetheless, this theory necessitates confirmation by targeted experiments. A potential option for future research involves optimizing the lipid component utilized in felt substrates by identifying natural sources of essential fatty acids homologous to the current formulation. Assessing these options may facilitate the development of a sustainable and practical pheromone treatment.

## 5. Conclusions

This study provides encouraging evidence regarding the potential of exogenous pheromone-related stimulation strategies to support the development of *Apis mellifera* colonies. The application of BEP and synthetic fatty acid blends (FABs), administered in association with a protein-rich diet, supported queen egg-laying activity and brood development under the experimental conditions tested.

Notably, the T3 fatty acid blend combined with protein feed generated responses comparable to, and in some cases slightly superior to, those observed with BEPs. These findings suggest that certain components of the larval pheromone signal may be partially mimicked by lipid analogs, highlighting the possible involvement of fatty acids with behavioral or regulatory roles at the colony level. The moderate effect observed in the T2 treatment indicates that, although fatty acids are essential components of bee nutrition, their supplementation within protein feed does not necessarily trigger an immediate stimulatory response, likely due to the colony’s capacity to regulate lipid intake according to its physiological needs.

However, these conclusions must be interpreted with caution. The relatively small number of experimental units represents an important limitation, restricting the generalizability of the findings. Consequently, the present work should be regarded as a preliminary study aimed at verifying whether the applied stimulatory formulations elicit the expected biological responses, before extending the approach to a larger number of colonies.

Despite these limitations, the results indicate that such interventions may contribute to supporting colony development during periods of nutritional stress or unfavorable climatic conditions. Future research involving larger sample sizes, multi-seasonal assessments, and longer monitoring periods is necessary to confirm the reproducibility and consistency of these effects. Expanding the experimental scale would also allow for a more robust evaluation of long-term impacts on colony performance and a clearer understanding of the mechanisms underlying pheromone–lipid interactions within the colony system.

## Figures and Tables

**Table 1:** Experimental treatments and their components.

Treatment	Pheromone Component/Baits	Food Component
Treatment 1	BEP *-rubber septa	solid food protein
Treatment 2	BEP *-rubber septa	solid food protein + FA ***
Treatment 3	FAB **-textile dispenser	solid food protein
Treatment 4	None (control)	solid food protein

* BEP—Synthetic brood pheromone applied on rubber septa, ** FAB—fatty acid blend on textile dispenser for stimulation, *** FA—unsaturated fatty acids incorporated in solid food protein.

**Table 2:** Chemical composition of volatile and semi-volatile compounds identified from honeybee brood by GC-MS.

No.	Identified Compounds	RT *	LRI **	SPME	Extract	Class Type
				%	%	
1.	1-Methyl-1-butene	4.63	791	0.52	-	alkene
2.	Octane	5.16	800	-	0.05	alkane
3.	Beta-ocimene	5.56	850	0.39	-	terpene
4.	2-Nonene	5.66	856	-	0.42	alkene
5.	1,1,2-Trichloro-ethane	5.87	860	-	0.41	halogen
6.	1-Butoxy-propanol	6.72	963	0.74	-	alcohol
7.	Methyl 3,4-dichlorobutanoate	7.31	890	-	0.47	ester
8.	Nonane	7.39	900	-	0.20	alkane
9.	Heptane, 3-ethyl-2-methyl	7.48	910	0.48	-	alkane
10.	Decene	8.14	950	-	0.41	alkene
11.	Methyl formate	8.30	960	0.66	-	ester
12.	1,1,2,2-Tetrachloro-ethane	9.00	1001	-	0.44	halogen
13.	Undecene	10.02	1058	-	0.07	alkene
14.	Benzoic acid methyl ester	10.42	1080	3.75	-	ester
15.	1-Dodecene	10.57	1088	-	0.26	alkene
16.	Cis-verbenone	11.43	1137	-	0.10	terpene
17.	1-Tridecene	12.80	1215	-	0.19	alkene
18.	2,2,4-trimethyl-1,3-pentanediol diisobutyrate	13.13	1234	4.15	-	ester
19.	Tridecane	14.24	1300	-	0.18	alkane
20.	Tetradecane	15.86	1400	-	0.51	alkane
21.	6-Undecylamine	16.02	1411	0.40	-	amine
22.	Methyl tetradecanoate	16.45	1439	-	1.80	ester
23.	Decanedioic acid dimethyl, ester	16.75	1459	-	0.13	ester
24.	2,4-Di-tert-butylphenol	17.25	1491	-	0.55	alcohol
25.	Pentadecane	17.38	1500	-	2.16	alkane
26.	Methyl palmitate	18.52	1579	-	0.56	ester
27.	Methyl oleate	18.79	1598	-	3.84	ester
28.	Benzoic acid	18.80	1599	0.39	-	acid
29.	Hexadecane	18.89	1600	-	4.44	alkane
30.	2-Oxanone	20.03	1688	2.16	-	ketone
31.	Methyl linoleate	20.08	1692	-	1.28	ester
32.	Heptadecane	20.19	1700	-	0.48	alkane
33.	Octadecane	21.49	1800	-	9.45	alkane
34.	n-Pentadecanoic acid	21.62	1812	0.57	-	acid
35.	Tridecane-7-hexyl	21.84	1833	-	8.20	alkane
36.	11-Buthyl-docosane	21.98	1846	-	1.14	alkane
37.	Nonadecane	22.56	1900	-	0.50	alkane
38.	Heneicosane	24.15	2100	-	14.67	alkane
39.	Palmitic acid	24.19	2106	5.62	-	acid
40.	1-Docosene	24.39	2137	-	0.59	alkene
41.	Palmitoleic acid	24.71	2186	-	0.42	acid
42.	Stearic acid	27.40	2612	3.82	1.10	acid
43.	Heptacosane	27.90	2700	-	6.98	alkane
44.	Octacos-1-ene	27.92	2702	-	2.37	alkene
45.	Oleic acid	28.01	2710	18.42	24.05	acid
46.	Linoleic acid	29.21	2816	57.88	1.06	acid
47.	Linolenic acid	31.11	3036	-	2.74	acid
48.	Nocason-1-ene	32.49	3045	-	7.70	alkene
	Total			99.95	99.92	

* RT—Retention time, ** LRI—Linear Retention Index.

**Table 3:** Colony egg-laying and brood area (mean ± SD) under treatments in Apiary 1 (SU).

Treatment	Variable	22 March 2023	3 April 2023	11 April 2023	20 April 2023
		Mean ± SD (dm^2^)	Mean ± SD (dm^2^)	Mean ± SD (dm^2^)	Mean ± SD (dm^2^)
T1	Egg-laying	3.67 ± 1.26	8.17 ± 1.04	14.83 ± 2.25	20.50 ± 2.50
	Brood area	13.17 ± 1.53	14.17 ± 1.04	22.33 ± 2.02	32.00 ± 2.00
T2	Egg-laying	1.00 ± 1.00	5.25 ± 0.43	8.83 ± 1.76	12.67 ± 1.53
	Brood area	13.67 ± 1.26	15.83 ± 1.76	19.83 ± 1.76	31.00 ± 2.00
T3	Egg-laying	1.83 ± 0.29	9.25 ± 0.90	18.33 ± 1.53	26.17 ± 2.25
	Brood area	12.50 ± 2.00	18.33 ± 2.02	27.33 ± 1.26	39.17 ± 1.26
T4	Egg-laying	10.17 ± 1.26	6.50 ± 1.32	9.17 ± 0.76	19.33 ± 1.61
	Brood area	3.33 ± 1.53	17.17 ± 2.25	20.17 ± 0.76	28.00 ± 1.00

Values are presented as mean ± standard deviation (SD), based on n = 3 replicates.

**Table 4:** Colony egg-laying and brood area (mean ± SD) under treatments in Apiary 2 (CA).

Treatment	Variable	22 March 2023	3 April 2023	11 April 2023	20 April 2023
		Mean ± SD (dm^2^)	Mean ± SD (dm^2^)	Mean ± SD (dm^2^)	Mean ± SD (dm^2^)
T1	Egg-laying	4.67 ± 1.53	11.83 ± 1.53	21.67 ± 2.08	28.33 ± 2.75
	Brood area	15.00 ± 2.00	17.50 ± 2.00	26.50 ± 2.00	36.67 ± 1.53
T2	Egg-laying	1.50 ± 1.00	7.00 ± 1.00	13.83 ± 1.53	19.50 ± 2.00
	Brood area	12.17 ± 1.53	15.50 ± 2.00	19.00 ± 2.00	30.00 ± 2.00
T3	Egg-laying	2.83 ± 1.26	16.5 ± 1.00	40.33 ± 1.53	51.00 ± 2.00
	Brood area	12.00 ± 2.00	22.33 ± 2.52	31.33 ± 2.52	43.33 ± 1.76
T4	Egg-laying	11.67 ± 1.53	8.00 ± 1.50	11.67 ± 1.76	24.67 ± 2.52
	Brood area	4.17 ± 1.53	21.00 ± 2.00	23.83 ± 2.08	28.33 ± 3.06

Values are presented as mean ± standard deviation (SD), based on n = 3 replicates.

## Data Availability

The original contributions presented in this study are included in the article/Supplementary Material. Further inquiries can be directed to the corresponding author.

## Supplementary Materials

The following supporting information can be downloaded at: https://www.mdpi.com/article/10.3390/insects17030294/s1, Table S1. *p*-values for Tukey’s HSD pairwise comparisons of egg laying in Apiary 1 at each evaluation date (corresponding to Figure 3). Table S2. *p*-values for Tukey’s HSD pairwise comparisons of brood area in Apiary 1 at each evaluation date (corresponding to Figure 4). Table S3. *p*-values for Tukey’s HSD pairwise comparisons of egg laying in Apiary 2 (corresponding to Figure 5). Table S4. *p*-values for Tukey’s HSD pairwise comparisons of brood area in Apiary 1 at each evaluation date (corresponding to Figure 6).

## Abbreviations

The following abbreviations are used in this manuscript:

SPME	Solid-phase microextraction
GC-MS	Gas chromatography coupled with mass spectrometry
RJ	Royal jelly
HPGs	Hypopharyngeal glands
MGs	Mandibular glands
BEPs	Brood ester pheromones
FAB	Fatty acid blend
FA	Unsaturated fatty acids
VOCs	Volatile organic compounds
RT	Retention time
LRI	Linear retention index
SD	Standard deviation
