# Supplementary material for: A New Approach Integrating Brood-Associated Semiochemicals with Additional Feeding for Honey Bee (Apis mellifera) Colony Development
Source: Insects. 2026 Mar 7;17(3):294. doi: 10.3390/insects17030294 (PMC13026789; doi:10.3390/insects17030294)
Supplement: Supplementary file 1 [file insects-17-00294-s001.zip › insects-4155134-supplementary.pdf]

Table S1. p-values for Tukey's HSD pairwise comparisons of **egg laying** in Apiary 1 at each evaluation date. (corresponding to Figure 3).

treatment / date	T2 vs T1	T3 vs T1	T3 vs T2	T4 vs T1	T4 vs T2	T4 vs T3
22.03.2023	0.052440	0.208770	0.759070	0.000259	0.000021	0.000042
3.04.2023	0.02683	0.55719	0.00460	0.23628	0.44770	0.03582
11.04.2023	0.0097	0.1210	0.0133	0.000519	0.9944	0.000663
20.04.2023	0.0026	0.0174	0.00006	0.8452	0.0070	0.0061

Table S2. p-values for Tukey's HSD pairwise comparisons of brood area in Apiary 1 at each evaluation date (corresponding to Figure 4).

treatment / date	T2 vs T1	T3 vs T1	T3 vs T2	T4 vs T1	T4 vs T2	T4 vs T3
22.03.2023	0.9796	0.9544	0.8090	0.000311	0.000220	0.000507
3.04.2023	0.6895	0.0888	0.3935	0.2598	0.8082	0.8603
11.04.2023	0.2625	0.0165	0.0014	0.3665	0.9928	0.0019
20.04.2023	0.8730	0.0029	0.0012	0.0654	0.1873	0.000141

Table S3. p-values for Tukey's HSD pairwise comparisons of egg laying in Apiary 2 (corresponding to Figure 5).

treatment / date	T2 vs T1	T3 vs T1	T3 vs T2	T4 vs T1	T4 vs T2	T4 vs T3
22.03.2023	0.0787	0.3978	0.6364	0.000979	0.000070	0.000197
3.04.2023	0.0014	0.0017	0.000011	0.0060	0.6150	0.000025
11.04.2023	0.0025	0.0000048	0.00000020	0.000491	0.4663	0.000000054
20.04.2023	0.0041	0.00000763	0.00000040	0.1965	0.0797	0.00000206

Table S4. p-values for Tukey's HSD pairwise comparisons of brood area in Apiary 1 at each evaluation date (corresponding to Figure 6).

treatment / date	T2 vs T1	T3 vs T1	T3 vs T2	T4 vs T1	T4 vs T2	T4 vs T3
22.03.2023	0.2816	0.2427	0.9994	0.000331	0.0025	0.0029
3.04.2023	0.6749	0.0927	0.0188	0.2633	0.0541	0.8689
11.04.2023	0.0119	0.0960	0.000518	0.4740	0.0960	0.0119
20.04.2023	0.0227	0.0227	0.000306	0.0066	0.7838	0.000132
